# Retained Metabolic Flexibility of the Failing Human Heart

**DOI:** 10.1161/CIRCULATIONAHA.122.062166

**Published:** 2023-05-18

**Authors:** William D. Watson, Peregrine G. Green, Andrew J.M. Lewis, Per Arvidsson, Giovanni Luigi De Maria, Håkan Arheden, Einar Heiberg, William T. Clarke, Christopher T. Rodgers, Ladislav Valkovič, Stefan Neubauer, Neil Herring, Oliver J. Rider

**Affiliations:** Oxford Centre for Magnetic Resonance Research (W.D.W., P.G.G., A.J.M.L., P.A., L.V., S.N., O.J.R.), University of Oxford, UK.; Department for Physiology, Anatomy and Genetics (P.G.G., N.H.), University of Oxford, UK.; Wellcome Centre for Integrative Neuroimaging, FMRIB, Nuffield Department of Clinical Neurosciences (W.T.C.), University of Oxford, UK.; Department of Cardiology, Oxford University Hospitals NHS Foundation Trust, UK (G.L.D.M.).; Clinical Physiology, Department of Clinical Sciences Lund, Lund University, Skåne University Hospital, Lund, Sweden (P.A., H.A., E.H.).; Wolfson Brain Imaging Centre (C.T.R.), University of Cambridge, UK.; Department of Cardiovascular Medicine (W.D.W.), University of Cambridge, UK.; Institute of Measurement Science, Slovak Academy of Sciences, Slovakia (L.V.).

**Keywords:** adenosine triphosphate, heart failure, magnetic resonance spectroscopy, metabolism

## Abstract

The failing heart is traditionally described as metabolically inflexible and oxygen starved, causing energetic deficit and contractile dysfunction. Current metabolic modulator therapies aim to increase glucose oxidation to increase oxygen efficiency of adenosine triphosphate production, with mixed results.

**Methods:**

To investigate metabolic flexibility and oxygen delivery in the failing heart, 20 patients with nonischemic heart failure with reduced ejection fraction (left ventricular ejection fraction 34.9±9.1) underwent separate infusions of insulin+glucose infusion (I+G) or Intralipid infusion. We used cardiovascular magnetic resonance to assess cardiac function and measured energetics using phosphorus-31 magnetic resonance spectroscopy. To investigate the effects of these infusions on cardiac substrate use, function, and myocardial oxygen uptake (MVo_2_), invasive arteriovenous sampling and pressure–volume loops were performed (n=9).

**Results:**

At rest, we found that the heart had considerable metabolic flexibility. During I+G, cardiac glucose uptake and oxidation were predominant (70±14% total energy substrate for adenosine triphosphate production versus 17±16% for Intralipid; *P*=0.002); however, no change in cardiac function was seen relative to basal conditions. In contrast, during Intralipid infusion, cardiac long-chain fatty acid (LCFA) delivery, uptake, LCFA acylcarnitine production, and fatty acid oxidation were all increased (LCFA 73±17% of total substrate versus 19±26% total during I+G; *P*=0.009). Myocardial energetics were better with Intralipid compared with I+G (phosphocreatine/adenosine triphosphate 1.86±0.25 versus 2.01±0.33; *P*=0.02), and systolic and diastolic function were improved (LVEF 34.9±9.1 baseline, 33.7±8.2 I+G, 39.9±9.3 Intralipid; *P*<0.001). During increased cardiac workload, LCFA uptake and oxidation were again increased during both infusions. There was no evidence of systolic dysfunction or lactate efflux at 65% maximal heart rate, suggesting that a metabolic switch to fat did not cause clinically meaningful ischemic metabolism.

**Conclusions:**

Our findings show that even in nonischemic heart failure with reduced ejection fraction with severely impaired systolic function, significant cardiac metabolic flexibility is retained, including the ability to alter substrate use to match both arterial supply and changes in workload. Increasing LCFA uptake and oxidation is associated with improved myocardial energetics and contractility. Together, these findings challenge aspects of the rationale underlying existing metabolic therapies for heart failure and suggest that strategies promoting fatty acid oxidation may form the basis for future therapies.

**Clinical Perspective**

**What Is New?**

- In nonischemic cardiomyopathy, the failing heart is flexible in metabolic substrate, able to increase uptake of fat depending on the prevailing metabolic conditions or when required under stress.
- In the failing heart in nonischemic cardiomyopathy, a switch to fat metabolism does not cause clinically overt hypoxia.
- Increased fatty acid metabolism in the failing, nonischemic heart improves myocardial energetics (PCr/ATP ratio) and increases contractility.

**What Are the Clinical Implications?**

- Therapies that target fat metabolism may form the basis of novel treatments in cardiomyopathy.

Regardless of the pathogenesis, most cardiac diseases eventually manifest in systolic dysfunction. Because delivery of adenosine triphosphate (ATP) to the myofilaments and membrane pumps is essential for normal contractile function, it follows that a reduction in ATP delivery could serve as the final common pathway that allows many different pathogeneses to manifest as the same end phenotype (ie, heart failure [HF] with reduced ejection fraction [HFrEF]).

As the only immediate source of energy for contraction and relaxation of the heart, cardiac ATP demand is very high. To keep up with this demand, the heart needs to produce ≈20× its own weight in ATP per day.^1^ In the resting state, the healthy heart achieves this by producing ATP from the oxidation of mainly fatty acids (60% to 90%), with a lesser contribution from glucose (10% to 40%), but exhibits remarkable flexibility to alter its substrate preference depending on prevailing metabolic and hemodynamic conditions. This allows for the natural fluctuations in substrate supply that occur with fasting, feeding, and exercise to be accommodated, and matches ATP production to demand. A pathological reduction in ATP production would necessarily lead to impaired cardiac function; as a corollary, promoting cardiac ATP production could be a potential therapy for HFrEF. In HFrEF, cardiac fatty acid oxidation (FAO) rates are lower^2^ across animal models of infarction,^3^ pacing-induced HF,^4^ and also in humans.^5,6^ This results from downregulated peroxisome proliferator-activated receptor α (PPARα) signaling, which reduces fatty acid transport and downregulation of fatty acid oxidizing enzymes.^7^ In animal models, this reduction is not accompanied by decreased fatty acid uptake,^8^ leading to triglyceride storage, nonoxidative metabolism, and lipotoxic species, which may contribute to HFrEF progression.^9^ In addition, myocardial insulin resistance reduces glucose uptake and pyruvate dehydrogenase (PDH) flux is decreased.^10^ As such, both glucose and FAO are decreased,^11–15^ but as FAO is reduced proportionally more, a relative “substrate switch” toward glucose metabolism is observed.^1^ It is believed that glucose is used initially in anaplerotic pathways for amino acid synthesis (necessary for the generation of left ventricular [LV] hypertrophy) rather than serving as a substrate for myocardial ATP production.^16^

On the premise that the failing heart appeared metabolically inflexible,^17–19^ and oxygen-starved,^20,21^ a major therapeutic focus in HFrEF has been to identify pharmacological strategies to promote myocardial glucose oxidation, providing a more oxygen-efficient ATP synthesis pathway (glucose has higher phosphate/oxygen ratio [P/O] than fat).^17^ This can be achieved by either stimulating glucose uptake^22^ or improving the coupling of glucose uptake to oxidation by increasing PDH flux. PDH activity also can be increased by inhibition of FAO, and partial FAO inhibitors^23–26^ have been shown in small studies to produce improvements in LV function^27^ and energetics^28^ in chronic ischemic HFrEF. However, this approach is limited by the intrinsically lower yield of ATP from glucose oxidation (≈3.5× lower than FAO), which, when combined with the reduced cellular uptake from insulin resistance in HFrEF,^29,30^ is unlikely to fully compensate for the loss of FAO, which has a higher molar ATP yield per carbon. Should the human failing heart be shown to be metabolically inflexible and have sufficient oxygen to perform FAO at stress, then this therapeutic approach would be brought into question.

## Methods

The study was approved by the National Research Ethics Service (REC reference 18/SC/0170) and conformed to the principles outlined in the Declaration of Helsinki. Written informed consent was obtained from all participants. Participants with HFrEF were recruited from HF clinics, cardiac magnetic resonance imaging lists, and other studies in which the participants had consented to future contact. The original data that support the findings of this study are available from the corresponding author upon reasonable request.

### Study Population

We recruited 20 volunteers with nonischemic HF and 10 healthy volunteers to undergo intravenous infusion that aimed to manipulate cardiac metabolic substrate supply. We used noninvasive cardiac magnetic resonance (CMR) and phosphorus-31 magnetic resonance spectroscopy (^31^P-MRS) to measure contractility, phosphocreatine (PCr)/ATP ratio (PCr/ATP), and creatine kinase (CK) flux. We recruited an additional 9 volunteers (5 of whom had undergone the CMR/^31^P-MRS protocol) scheduled to have cardiac resynchronization therapy pacemaker insertion to undergo additional invasive measurements of cardiac oxygen and substrate use (arteriovenous sampling) and LV function with pressure–volume loop interrogation at the time of their device implant. All participants were studied after an overnight fast.

### Inclusion Criteria for Participants With HF

Participants had symptomatic HF as defined by European Society of Cardiology guidelines^31^ and LV ejection fraction (LVEF) <40% by echocardiography or CMR.

### General Exclusion Criteria

Exclusion criteria were diabetes medications, egg or soy allergy, disturbances of normal fat metabolism (eg, hyperlipidemia, lipoid nephrosis, or pancreatitis), liver disease (eg, imaging evidence of cirrhosis or liver function tests outside the normal range), recent fracture of pelvis or long bones, anemia (defined as hemoglobin below the normal range), blood coagulation disorders (not including the use of oral anticoagulants), or metallic foreign bodies preventing CMR imaging.

### Exclusion Criteria for Participants With HF

Exclusion criteria were ischemia as primary cause of HFrEF (no infarction in one or more segments on CMR or not greater than mild coronary artery disease on coronary angiography, as defined by 50% luminal stenosis).

### Exclusion Criteria for Healthy Volunteers

Exclusion criteria were any LV dysfunction or other cardiac diagnosis.

### Cardiac Substrate Manipulation

#### To Increase Cardiac Fatty Acid Supply

To elevate free fatty acid supply, 20% Intralipid (Fresenius Kabi) was infused intravenously at 60 mL per hour. For those not taking anticoagulant medications, unfractionated heparin (Monoparin; CP Pharmaceuticals) was concomitantly infused at 0.4 U/kg per hour to increase lipolysis. This was infused for 1 hour before scanning in the CMR arm, or commenced 15 minutes before measurements in the invasive arm, and then continued throughout the subsequent experiment.

#### To Increase Cardiac Glucose Supply

To increase cardiac glucose, a euglycemic hyperinsulinemic clamp was used. Insulin (Actrapid; Novo) was infused at 0.8 mU/kg per minute with an infusion of 20% glucose titrated to maintain blood glucose measurements at around the baseline for participants. Samples of venous blood were taken from a cannula up to every 5 minutes and tested with a point-of-care device (Freestyle Optimum Neo; Abbott). Patients were established on the euglycemic clamp for 1 hour in both arms before study measures were obtained.

### Noninvasive Imaging Protocol

All participants underwent the after-CMR imaging protocol. All scans were performed at 3T (Tim Trio; Siemens Healthineers). At the first visit, all participants were established on either an insulin+glucose (I+G) or Intralipid infusion 1 hour before CMR imaging, with the infusion type determined randomly by a computer-generated algorithm. Venous blood samples were drawn immediately before scanning. The noninvasive protocol was repeated after 7 days with the other infusion.

#### LV Imaging

A short stack of cine images was obtained using a 24-channel spine matrix coil, 6-channel body array coil (both Siemens), and steady-state free precession sequences, as described previously.^32^ In brief, all images were ECG-gated and taken during end-expiratory breath-hold. Typical steady-state free precession sequence measures were: slice thickness, 8 mm; gap, 2 mm; retrospective gating, 1.5 ms; repetition time (TR), 46 ms; flip angle 50º; field of view, 400 mm; and matrix size, 256 in frequency encode direction. When necessary, prospective gating was used in atrial fibrillation. Images were analyzed with manually drawn LV endocardial and epicardial contours in cvi42 (Circle Cardiovascular Imaging), as described previously.^33^ The validation of CMR-derived pressure–volume loops from CMR and blood pressure is described separately in the Expanded Methods in the Supplemental Material.

### Cardiac ^31^P-MRS

#### Myocardial PCr/ATP

Participants were placed into a prone position over a 3-element, dual-tuned ^1^H/^31^P surface coil at magnet isocenter (2× ^31^P Tx/Rx loops, 1× ^1^H Tx/Rx loop; Siemens Healthcare). A nongated 3D acquisition-weighted ultrashort echo time chemical shift imaging sequence was run as described previously.^33^ Parameters included: acquisition matrix size 16 × 8 × 8 voxels, field of view 240 × 240 × 200 mm^3^, nominal voxel size 11.25 mL, 10 averages at the center of k-space, TR per subject 910–1010 ms. The PCr/ATP ratio reported is the blood and saturation–corrected PCr/average ATP ratio, averaged over the 2 most basal septal voxels. Spectral analysis was performed using OXSA toolbox, an open-source MATLAB implementation of the AMARES algorithm.^34^

#### Myocardial CK Forward Rate Constant

The pseudo-first order unidirectional rate constant (CK forward rate constant [CK kf]) was estimated using triple TR saturation transfer adapted for use with a transmit/receive 10-cm loop radiofrequency coil (PulseTeq Ltd) as described previously.^35^ In brief, participants were scanned supine, ^1^H localizers confirmed coil position, and a 1-dimensional phase-encoded chemical shift imaging matrix (16 slices; 160 mm) was used to acquire 4 sets of ^31^P spectra: a fully relaxed acquisition (TR 15 seconds; 2 averages), 2 acquisitions with selective γATP saturation (TR, 1.5 then 9.5 seconds), and 1 acquisition with control saturation mirrored around PCr (TR, 15 seconds, 2 averages). Spectral analysis was performed using custom software as described previously.^35^ CK k_f_ in the ATP-generating (forward) direction was then calculated as:


$$\begin{array}{l} k_{f}^{CK}=\frac{1}{T_{1}^{*}}\left(\frac{M_{PCr}^{Ctrl}}{M_{\text{PCr}}^{\prime }}-1\right). \end{array}$$

Forward CK flux was calculated by [PCr]×k_f_, where [PCr] is estimated by multiplying PCr/ATP by literature values^36^ for [ATP] in HFrEF (5.2 μmol/g).

#### Increased Workload

After the resting CK k_f_ measurement was taken, dobutamine was infused to increase cardiac work (targeted to 65% of maximum predicted for age). After target heart rate was achieved, cine images were again acquired for cardiac function, and 2 further ^31^P spectra were acquired (selective γATP saturation and control saturation); CK k_f_ during stress was then calculated as described previously.^35^

### Invasive Catheter Protocol

All measurements were taken during scheduled cardiac resynchronization therapy pacemaker implantation. Before the procedure, all patients were established on a euglycemic hyperinsulinemic clamp as described previously. After the implantation of atrial and ventricular leads, radial and femoral arterial access was established. A guide catheter was passed to the left main coronary artery through the right radial approach, and a flow wire (Volcano; Philips Healthcare) was passed into the proximal segment of the left main coronary artery to measure coronary arterial flow velocity (cm/s). Coronary flow (mL/s) was then calculated as π×coronary radius^2^ (taken from fluoroscopy)×flow. Through a femoral arterial approach, a conductance catheter (Inca; Leycom) was passed across the aortic valve into the left ventricle, and pressure–volume measurements were taken. At each measurement, 60 seconds of recordings from the impedance catheter and the flow wire were taken, along with paired arterial (from the coronary artery catheter) and venous blood sampling (from the coronary sinus). This was performed during intrinsic rhythm, and again during rapid atrial pacing (65% maximal heart rate for age, determined at 220−age).

After these measurements were performed, the I+G infusion was stopped, and an infusion of Intralipid was started (as described above). Fifteen minutes after the beginning of the Intralipid infusion, the sampling protocol outline was repeated during intrinsic rhythm, and atria–atria–interrupt pacing (AAI; a pacing mode where the atrium is paced) was undertaken. Blood samples were assayed for oxygen saturation, partial pressure of oxygen, and hemoglobin using a blood gas analyzer (Seca). Glucose, nonesterified free fatty acids, β-hydroxybutyrate, insulin, and lactate levels were all assessed in the clinical-grade Oxford University Hospitals Clinical Laboratories.

Myocardial oxygen usage (MVo2) was determined from arteriovenous content difference (0.0225×Po2 KPa+1.4×[Hgb]×[%O2 sat])×flow rate [mL/s]), as detailed previously. Myocardial carbon dioxide production (MVco_2_) was calculated from the McHardy equation:

ΔCO_2_=11.02×[(Pvco_2_)0.396–(Paco_2_)0.396]–(15–Hb)×(Pvco_2_–Paco_2_)–(95–Sao_2_)×0.0064. Respiratory quotient (RQ) was calculated as cardiac Vco_2_/Vo_2_. Myocardial substrate uptake was determined from arteriovenous difference (glucose/lactate/ketone/nonesterified free fatty acids)×coronary flow rate. Metabolomics were run by a commercial provider (Metabolon) and analyzed as described in the Expanded Methods in the Supplemental Material.

### Statistics

Statistical analysis was performed using GraphPad Prism (GraphPad Software). All data were subjected to Kolmogorov‐Smirnov tests to establish normal distribution and are presented as mean±SD unless otherwise stated. Paired Student *t* tests were performed for paired data sets and repeated-measures ANOVA were performed for multiple data sets. Correlations were assessed using Pearson correlation analysis. All metabolomic and lipidomic analysis was subject to false discovery rate (set at 0.05) analysis using the Benjamini-Hochberg correction. A probability of *P*<0.05 was considered significant (2-tailed).

## Results

### Healthy Volunteers

Baseline demographics are shown in Table S2, and the results of substrate manipulation are shown in Figure S2. Intralipid caused an elevation in circulating nonesterified fatty acids compared with the I+G infusion (3.06±0.65 versus 0.13±0.1 mmol/L; *P*<0.0001), accompanied by a higher LVEF (63±3.4 versus 58.1±3.8; *P*=0.001). PCr/ATP ratio was not significantly different (1.98±0.34 versus 2.05±0.3; *P*=0.57), but CK first-order rate constant was elevated (0.34±0.13 versus 0.21±0.09/s; *P*=0.02), with a trend to an increased CK flux (3.45±1.48 versus 2.37±0.93 µmol/g per second; *P*=0.05).

### HF Volunteers

#### Anthropometric and Baseline Study Data

Baseline data for the cohorts are shown in the Table. When comparing the invasive and noninvasive HFrEF cohorts, there were no significant differences in any baseline measure. Median New York Heart Association classification was II, and median baseline ejection fraction was 35% in both groups. All patients were on maximum tolerated doses of goal-directed medical therapy for HF.

**Table.**
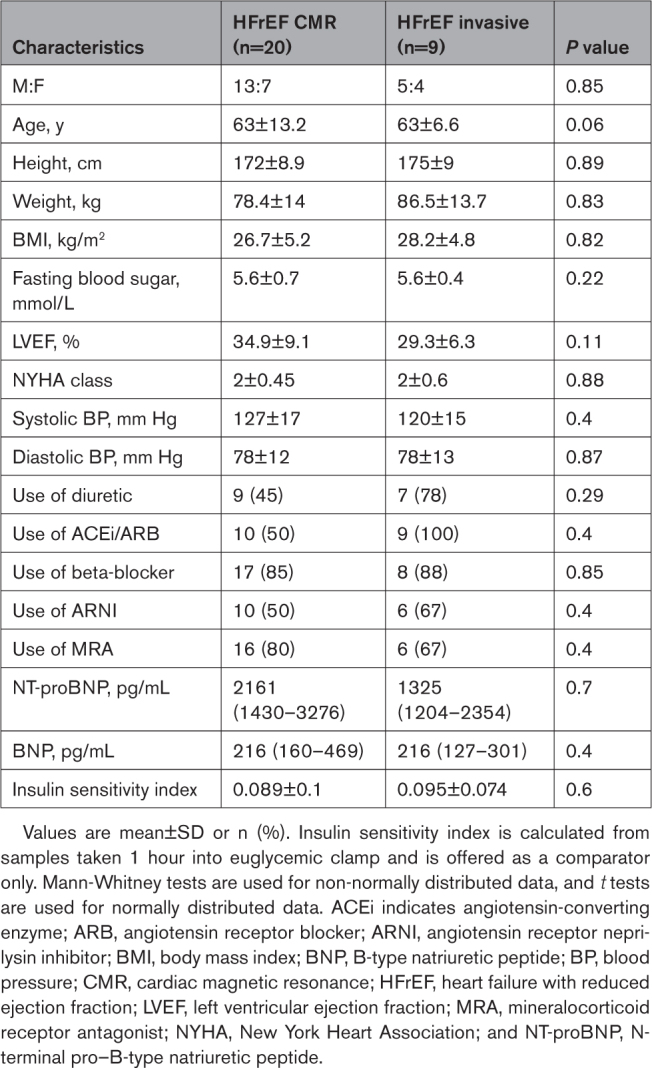
Baseline Data for the Noninvasive and Invasive Cohorts

#### Retained Substrate Flexibility

**Figure 1.**
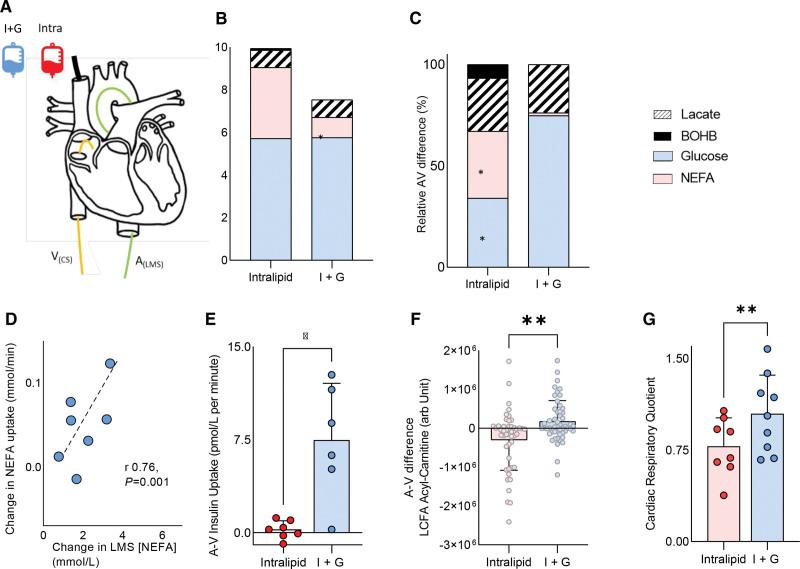
**Assessment of cardiac substrate flexibility during altered substrate supply. A**, Schematic of the experimental setup. **B**, Left main stem (LMS) arterial substrate concentrations. **C**, Relative arteriovenous (AV) difference of nonesterified free fatty acids (NEFA), glucose, lactate, and β-hydroxybutyrate (BOHB). **D**, Correlation between LMS NEFA concentration and uptake. **E**, Cardiac insulin uptake. **F**, Long-chain fatty acid (LCFA) acylcarnitines. **G**, Cardiac respiratory quotient (*P<0.05; **P<0.01; ***P<0.001). Data are presented as mean with SD error bars unless stacked. A_(LMS)_ indicates arterial left main stem sample; and V_(CS)_, venous coronary sample.

**Figure 2.**
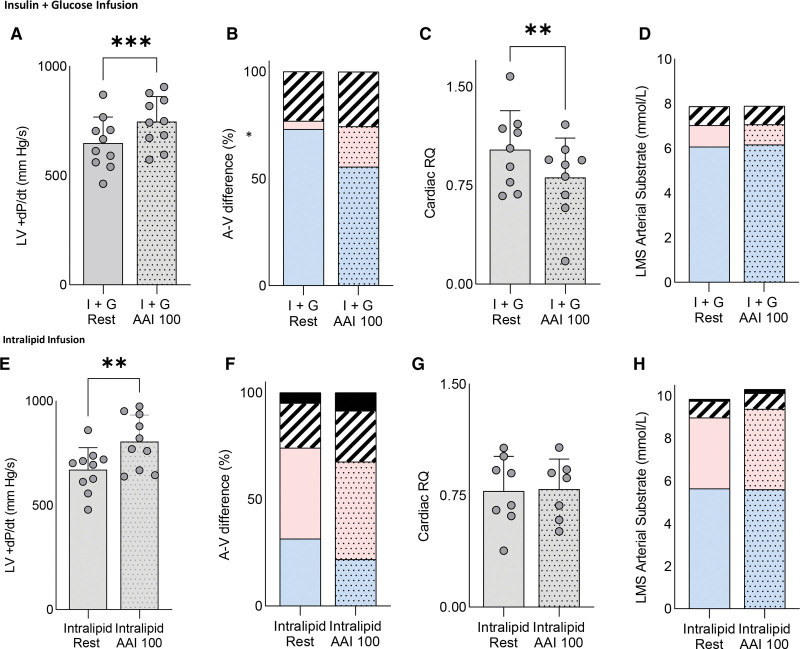
**Assessment of cardiac substrate flexibility during increased workload with insulin+glucose infusion and Intralipid infusion.** Assessment of cardiac substrate flexibility during increased workload (atria–atria–interrupt pacing 100 beats per minute [AAI 100]) during insulin+glucose (I+G) infusion (**A** through **D**) and Intralipid infusion (**E** through **H**) detailing relative arteriovenous difference of nonesterified free fatty acids, glucose, lactate, β-hydroxybutyrate (BOHB), left ventricle (LV)+change in pressure/change in time (dp/dt), and cardiac respiratory quotient (RQ). Spotted fill indicates stress measurement (*P<0.05; **P<0.01; ***P<0.001). Data are presented as mean with SD error bars unless stacked. In stacked charts, blue represents lipid; red, glucose; stripes, lactate; and black, ketone. LMS indicates left main stem.

**Figure 3.**
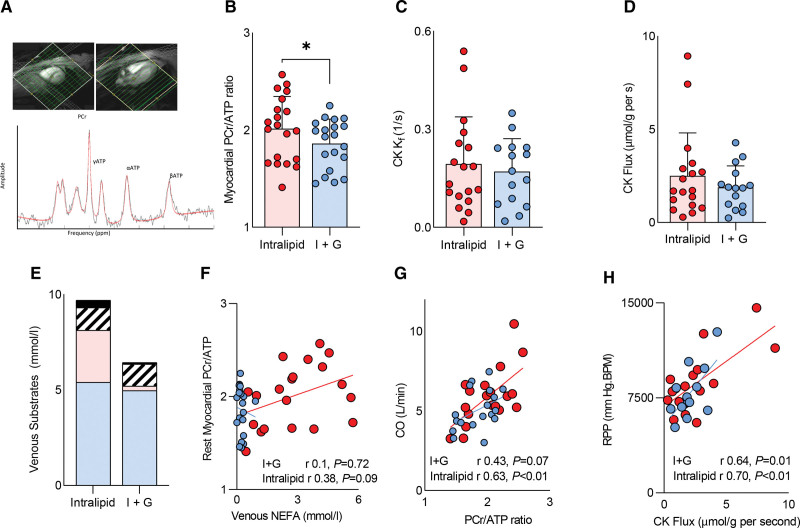
**Assessment of myocardial energetics at rest. A**, ^31^P magnetic resonance spectroscopy. **B**, Phosphocreatine/adenosine triphosphate ratio (PCr/ATP). **C**, Creatine kinase (CK) first-order rate constant (CK k_f_). **D**, CK flux. **E**, Venous circulating substrates. **F**, The relationship between PCr/ATP and venous fatty acid (nonesterified free fatty acids [NEFA]) and cardiac output (CO; **G**). **H**, The relationships between CK flux and rate pressure product (RPP). Spotted indicates stress measurement (*P<0.05). Data are presented as mean with SD error bars. I+G indicates insulin+glucose.

When left main stem arterial long-chain fatty acid (LCFA) supply was increased by Intralipid infusion (Figure 1A and 3E), myocardial LCFA uptake was higher, and glucose uptake was >2-fold lower than during I+G infusion (both *P*<0.05; Figure 1C). At rest, cardiac uptake was related to arterial supply for both LCFA (*r*=0.74; *P*<0.001; Figure 1D) and glucose (*r*=0.43; *P*=0.06). As expected, insulin was only taken up by the heart during I+G infusion (Figure 1E). In addition, metabolomic analysis of the arteriovenous samples showed that long-chain fatty acylcarnitines were produced by the heart only during Intralipid infusion (Figure 1F; *P*<0.01). Furthermore, cross-heart RQ was significantly lower during Intralipid than during I+G infusion (0.78±0.2 versus 1.0±0.3; *P*<0.01), showing a change in substrate oxidized (Figure 1G). The failing heart also displayed clear substrate flexibility when workload was changed. During I+G infusion and AAI pacing (100 bpm; Figure 2A), the increased workload (Figure 2A) was associated with a 70% increase in LCFA uptake (*P*=0.05; Figure 2B) and increased FAO (RQ reduced from 1.0±0.3 to 0.81±0.3; *P*<0.01; Figure 2C), despite no change in supply, and low levels of circulating LCFA (Figure 2D). During Intralipid infusion, AAI pacing again increased workload (Figure 2E) but did not change substrate supply or use significantly (Figure 2F through 2H). However, there was a trend to further increase ketone uptake (by 74%; *P*=0.12). Overall, this shows that during changes in both arterial substrate supply and cardiac workload, substrate flexibility is surprisingly retained in compensated nonischemic HF with severe LV systolic dysfunction.

#### Myocardial Energetics During Substrate Manipulation

Compared with I+G, Intralipid infusion was associated with an increase in myocardial PCr/ATP ratio (1.86±0.25 versus 2.01±0.33; *P*=0.016; Figure 3A and 3B). When combined with a numerical increase in the CK k_f_ (by 13%; Figure 3C), myocardial ATP delivery was in absolute terms higher (CK flux by 37%), but this was not statistically significant (*P*=0.25; Figure 3D). Myocardial PCr/ATP ratio was related to both venous substrate concentrations during infusions (Figure 3E and 3F) and cardiac output (Figure 3G). As expected, CK flux was related to rate pressure product at rest (*r*=0.67; *P*<0.001; Figure 3G). When ATP production was calculated from the combination of cardiac substrates used^37^ (assuming oxidative metabolism, and no storage), this was >2-fold higher during Intralipid, with a larger percentage contribution to ATP production from LCFA (85±14 versus 27±23%; *P*<0.01). During dobutamine stress (see Figure S3), mean heart rate was 103±9 bpm on Intralipid and 103±8 bpm on I+G, myocardial phosphocreatine was not depleted, and PCr/ATP ratio, CK k_f_, and CK flux were not different between infusions (PCr/ATP 1.78 versus 1.77; *P*=0.6). Myocardial energetics during stress were again related to venous substrate concentrations, but, in contrast to the resting state, CK flux was related to glucose (r=0.72; *P*=0.001) and lactate (*r*=0.5; *P*=0.04) concentrations. No difference in rate pressure product was seen between infusions. This suggests that in the resting state, increased FAO is associated with improvement in myocardial energetics of the failing human heart and that during stress, ATP flux in the failing heart is also related to glucose and lactate supply.

#### Cardiac Functional Change With Substrate Manipulation

**Figure 4.**
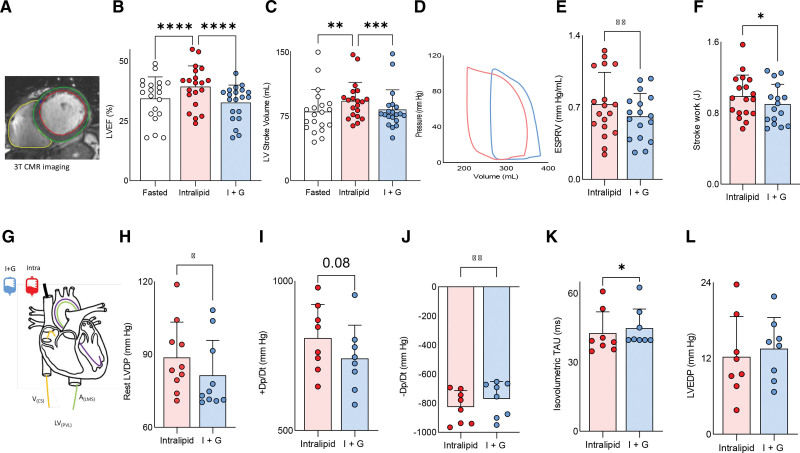
**Substrate manipulation and ventricular function at rest. A**, Contouring. **B**, Left ventricular ejection fraction (LVEF). **C**, Stroke volume. **D**, An example pressure–volume loop. **E**, End systolic pressure–volume relationship (ESPVR). **F**, Stroke work. **G**, A graphic of the invasive experiment. **H**, Left ventricular developed pressure (LVDP). **I**, change in pressure/change in time (+dp/dt). **J**, -Dp/Dt. **K**, Isovolumetric tau. **L**, Left ventricular end diastolic pressure (LVEDP; *P<0.05; **P<0.01; ***P<0.001; ****P<0.0001). Data are presented as mean with SD error bars. Throughout, Intralipid data in red; insulin+glucose (I+G) data, in blue. A_(LMS)_ indicates arterial left main stem sample; CMR, cardiac magnetic resonance; LV_(PVL)_, left ventricle (pressure volume loop); and V_(CS)_, venous coronary sample.

**Figure 5.**
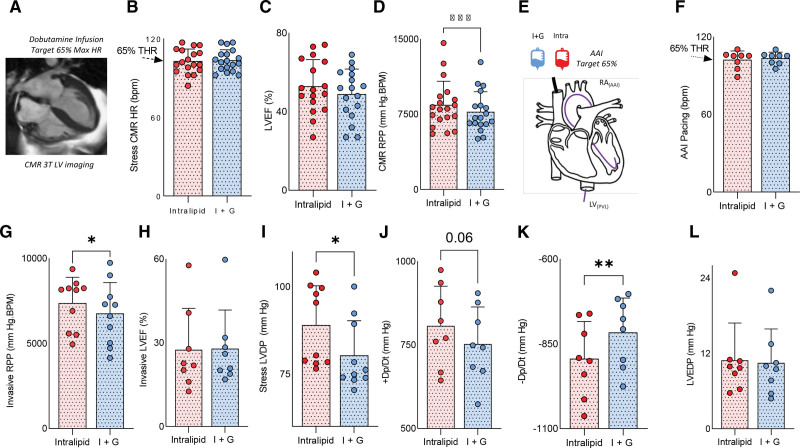
**Substrate manipulation and ventricular function during stress. A**, An example cardiac magnetic resonance (CMR) image. **B**, Dobutamine heart rate (HR), 65% target HR (THR) is shown. **C**, Left ventricular (LV) ejection fraction (LVEF). **D**, Rate pressure product (RPP). **E**, A graphic of the invasive stress experiment. **F**, HR during atria–atria–interrupt (AAI) pacing. **G**, RPP during AAI. **H**, LVEF by pressure–volume loop. **I**, Stress LVDP. **J**, Change in pressure/change in time (dp/dt). **K**, −dp/dt. **L**, LV end diastolic pressure (LVEDP; *P<0.05; **P<0.01; ***P<0.001; ****P<0.0001). Data are presented as mean with SD error bars. LV_(PVL)_ indicates left ventricle (pressure volume loop); and RA, right atrium.

Compared with fasted conditions and I+G infusion (during which no change in LVEF was seen), Intralipid infusion led to increases in LV contractile work on both noninvasive CMR (LVEF increase by 6.3%, LV stroke volume, stroke work, and end-systolic pressure–volume relationship; all *P*<0.05; Figure 4A through 4F) and invasive measurement (LV developed pressure [LVDP] by 8 mm Hg; *P*<0.05; change in pressure/change in time [dp/dt], by +6%; *P*=0.08; Figure 4H and 4I). At rest, diastolic function as assessed by LV −dp/dt and LV isovolumetric tau were also improved during Intralipid infusion (both *P*<0.05; Figure 4J and 4K). These improvements in systolic and diastolic function were seen without change in LV end-diastolic pressure (*P*=0.27; Figure 4L) or systolic blood pressure (baseline, 125±17; I+G, 119±20; Intralipid, 122±20 mm Hg; *P*=0.27). A similar pattern was seen during stress (Figure 5A through 5L). During Intralipid and AAI pacing, greater LVDP (by 10 mm Hg; *P*=0.04; Figure 5I) and +dp/dt (*P*=0.06; Figure 5J) were recorded. Stress diastolic function was also improved compared with I+G infusion (−dp/dt; *P*<0.01; Figure 5K), again without difference in LV end diastolic pressure (Figure 5L). This suggests that, at least in the short term, promoting FAO improves contractile function.

#### Myocardial Oxygen Use With Substrate Manipulation

**Figure 6.**
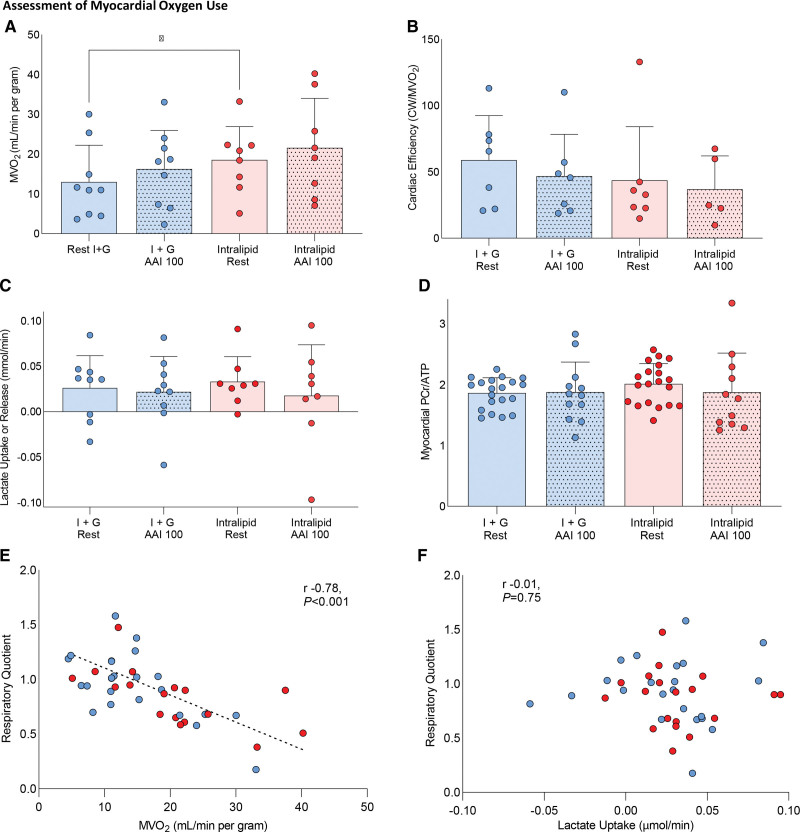
**Assessment of the effects of substrate manipulation and variable workload on myocardial oxygen use. A**, Myocardial oxygen use (MVo_2_ mL/min per gram). **B**, Cardiac efficiency (cardiac work/MVo_2_) changes. **C**, Cardiac lactate uptake. **D**, Myocardial phosphocreatine/adenosine triphosphate ratio (PCr/ATP) shown for the groups, along with the relationship between respiratory quotient and MVo_2_ (**E**) and myocardial lactate uptake (**F**). Dotted fill pattern demotes acquired during stress (*P<0.05). All analyses are repeated-measures ANOVA with Bonferroni correction. Correlation statistics are for Pearson R. Dotted fill denotes stress (atria–atria–interrupt [AAI] or dobutamine); plain denotes rest. Data presented as mean with SD error bars.

During Intralipid in the resting state, MVo_2_ was increased by 27% (*P*<0.05; Figure 6A) compared with I+G infusion, likely reflecting the inherently lower P/O ratio of FAO and increased contractile function. As a result, MVo_2_ at rest during Intralipid was similar to that seen during rapid AAI pacing during I+G infusion, and there was a trend to reduced myocardial oxygen efficiency during Intralipid infusion (Figure 6B). Even during the highest MVo_2_ data set (achieved during rapid pacing and Intralipid infusion), net cardiac lactate consumption still occurred (Figure 6C), and mean RQ recorded was 0.83, both showing aerobic metabolism. In addition, during dobutamine infusion, no depletion of phosphocreatine occurred (Figure 6D). Furthermore, as MVo_2_ increased, RQ was observed to decrease, for both infusions, suggesting that as workload increased, there was a preference to oxidize fatty acids (Figure 6E), with no relationship between lactate uptake and RQ seen (Figure 6F). In summary, the combination of increased MVo_2_, stable phosphocreatine pool, and lactate consumption are evidence that mitochondrial oxygen supply was not limited, even during high workloads.

#### Intermediary Metabolism

**Figure 7.**
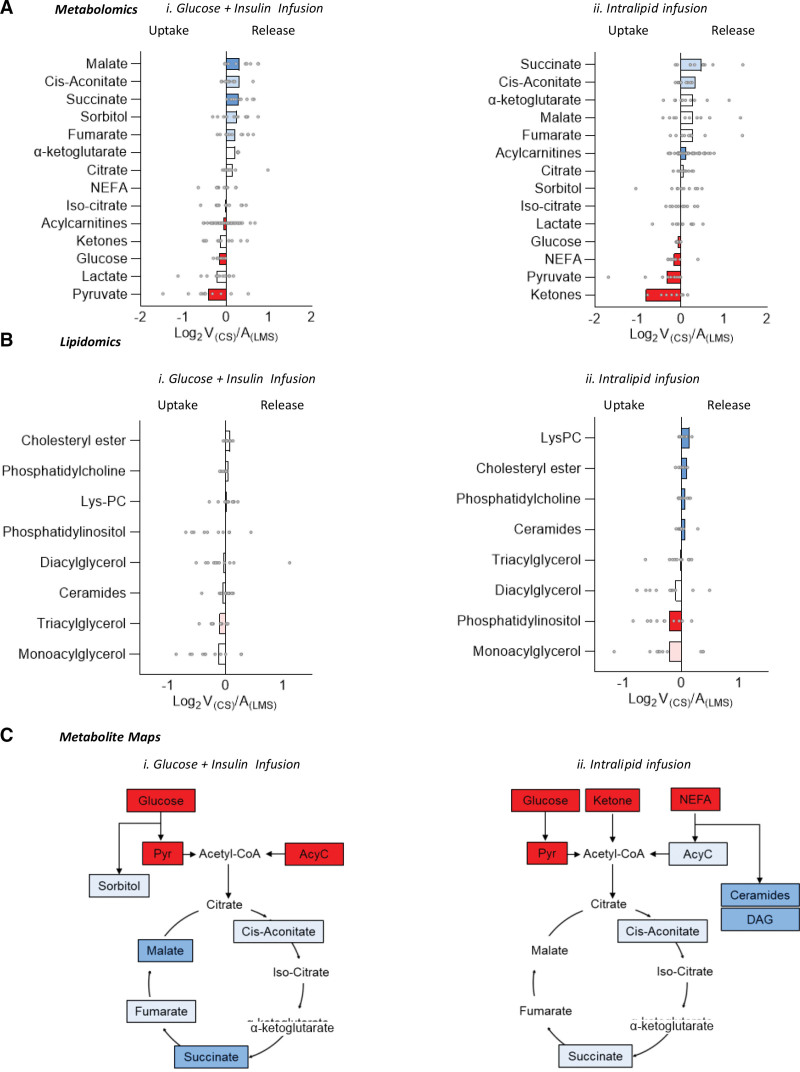
**Arteriovenous metabolomics of cardiac uptake and release of substrates and tricarboxylic acid cycle intermediates, arteriovenous lipidomic assessment, and metabolic maps demonstrating the relative position in the tricarboxylic acid cycle of influxed and effluxed metabolites during insulin+glucose infusion and Intralipid infusion. A**, Arteriovenous metabolomics of cardiac uptake/release of substrates and tricarboxylic acid cycle intermediates during (i) insulin+glucose (I+G) infusion and (ii) Intralipid infusion. **B**, Arteriovenous lipidomic assessment during (i) I+G infusion and (ii) Intralipid infusion. **C**, Metabolic maps demonstrating the relative position in the tricarboxylic acid cycle of influxed/effluxed metabolites during (i) I+G infusion and (ii) Intralipid infusion. Red denotes cardiac uptake (dark red, *P*<0.05; light red, *P*<0.1); blue, cardiac release (dark blue, *P*<0.05; light blue, *P*<0.1). All *P* values are adjusted for false discovery rate with Benjamini-Hochberg correction. A_(LMS)_ indicates arterial left main stem sample; LysPC, lysophosphatidylcholine; NEFA, nonesterified free fatty acid; and V_(CS)_, venous coronary sample.

Although we did not directly assay cellular metabolism, inferences about usage or production can be made from arteriovenous differences in intermediary metabolites. Alternative substrate sources, tricarboxylic acid cycle intermediates, and toxic intermediate metabolite production were assessed using metabolomic and lipidomic analysis of paired cardiac arteriovenous samples (ie, between left main stem coronary artery and coronary sinus). In addition to pyruvate and glucose uptake, which was seen during both infusions (Figure 7Ai and 7Aii), both ketones and fatty acids were taken up during Intralipid infusion. In line with the observed switch toward FAO during Intralipid, long-chain acylcarnitines were produced by the heart during Intralipid and taken up by the heart during I+G infusion. Branched-chain amino acids (leucine, isoleucine, and valine) were not altered by infusion type, and arteriovenous extraction remained similar (data not shown). Tricarboxylic acid cycle intermediates followed a similar pattern of uptake and release during both infusions (Figure 7Ai and 7Aii).

Evidence of toxic intermediate production was seen during both infusions, with evidence of the polyol pathway and sorbitol release being seen only during I+G infusion (*P*=0.052), and nonoxidative metabolism of fatty acids seen with ceramide release only during Intralipid infusion (*P*=0.03). Release of lysophosphatidylcholine, phosphatidylcholine, and cholesteryl ester was seen during Intralipid infusion but not during I+G infusion (Figure 7Bi and 7Bii).

Although the failing heart in HFrEF displays significant flexibility in substrate source to produce acetyl-CoA (coenzyme A), no major changes in the pattern of tricarboxylic acid cycle intermediate production or use are seen. However, the excess delivery of fatty acids or glucose used here to manipulate substrate use is associated with toxic nonoxidative metabolism and ceramide and sorbitol production, respectively. Metabolic maps are shown in Figure 7C.

## Discussion

On the basis of the hypothesis that the failing heart is metabolically inflexible, and fatty acid metabolism creates additional oxygen demand, current metabolic therapies aim to increase glucose oxidation due to its inherently greater oxygen efficiency. In this study, we have shown that the heart, in compensated nonischemic HF with severe LV systolic dysfunction, is not metabolically rigid, and retains considerable flexibility to change substrate uptake and metabolism according to changing arterial substrate supply and workload. In addition, we show that although increasing fatty acid uptake and oxidation is associated with improved myocardial energetics and positive inotropy, no change is seen with increased glucose oxidation. Furthermore, we show that even during increased FAO under stress protocols, despite increased myocardial oxygen usage, myocardial phosphocreatine pool is not depleted, and lactate is consumed, suggesting that the oxygen supply is adequate to match oxidative phosphorylation. Taken together, this challenges the existing doctrine in HF metabolism and suggests increasing FAO may be a beneficial therapeutic intervention.

### Metabolic Flexibility

The failing heart is traditionally described as metabolically inflexible, unable to change substrates when prevailing conditions change, unlike in the healthy heart.^38^ This conclusion is derived from a body of literature showing reduced fatty acid and glucose oxidation, but no previous study has altered cardiac substrate supply in HFrEF to investigate metabolic flexibility.

To demonstrate metabolic substrate flexibility, we exposed the heart to changes in workload and substrate supply. These may be considered a form of metabolic stress test. To overcome inhibition at CPT-1 (carnitine palmitoyltransferase 1; for LCFA) and insulin resistance (for glucose), both infusions were performed to achieve supraphysiological arterial supply and uptake levels.

Using these infusions, we demonstrated that considerable substrate flexibility is retained, with a change in LCFA and glucose uptake and oxidation being induced by a change in arterial substrate supply. We showed that LCFA plasma membrane uptake is associated with production of long-chain fatty acylcarnitines, revealing that LCFA substrates for oxidation are being transported into the mitochondria. The cross-heart respiratory quotient dropped to 0.78 during Intralipid infusion, confirming that the heart is increasing FAO.

As expected, the heart is able to take up ketone, with both β-hydroxybutyrate and aceto-acetate uptake being observed during Intralipid infusion, and (as expected) no circulating ketone was detected during I+G infusion.

### Change in Workload

Although physiological exercise would have been the ideal choice to investigate the effects of increased cardiac work, to limit movement artefact during ^31^P-MRS acquisition, dobutamine infusion was used. In contrast, as sequential infusions were used during the invasive experiment, a stress modality was required that could be started and stopped, and without time residual effects (as seen with both dobutamine and exercise). This necessitated rapid atrial pacing to be used as the stress modality for the invasive studies.

It has been shown previously in HFrEF that rapid AAI pacing increases FAO in the fasted state, and in contrast that the normal heart increases glucose oxidation.^11^ Our study adds that during rapid atrial pacing and I+G infusion, cardiac extraction of fatty acid increased >5-fold despite no change in arterial supply, with glucose uptake being numerically but not significantly reduced (by 24%). This occurred without a change in arterial supply, implying that the heart in HFrEF has active substrate selection and preference to fatty acids during increased workload.

### Cardiac Energetics

To assess whether substrate flexibility influenced cardiac high-energy phosphate metabolism, we used ^31^P-MRS to measure the PCr/ATP ratio, a sensitive index of the energetic status of the heart, and saturation transfer ^31^P-MRS to measure CK kinetics. Here, we show that myocardial PCr/ATP ratio was returning toward normal during Intralipid infusion in participants with HF. This contrasts with the healthy volunteer group, for whom it was unchanged and presumably already maximal. When put together with the numerical (although not statistically significant) 37% increase in ATP delivery rate through CK, this suggests that myocardial energetics are improved during oxidation of fatty acids when compared with glucose. This is in line with a greater molar ATP yield (≈3.5×) of fatty acids than glucose. The healthy volunteer group mounted a similar increase in CK flux, driven in their cases by a rise in CK rate constant.

The concentration of phosphocreatine relative to ATP rose and CK k_f_ did not fall during Intralipid, strongly implying that myocardial ATP supply did not fall below mitochondrial ATP demand during a period of increased contractility. Given the increased death rate of the chronic use of inotropes such as dobutamine and adrenaline^39^ due to adverse remodeling and energetic mismatch,^40^ this is an important observation.

### Cardiac Function

On both invasive and noninvasive measures of cardiac function compared with I+G, when no change from basal condition was observed, Intralipid infusion was associated with a measurable increase in contractility, with improvements in LVEF, LV strain, LV stroke work, LV +dp/dt, and LVDP all being observed. Healthy volunteers showed similar increases. LV end diastolic pressure was not different between groups, indicating that loading conditions could not have played a role in increasing contractility. As cardiac contractile function is a highly energetically demanding process, and FAO provides more ATP per mole oxidized, it is likely that increases in ATP production (as evidenced by a higher PCr/ATP ratio and CK flux during Intralipid) are at least in part responsible for improved contractility. This is seen here, with the strong positive correlation between measured CK flux and rate pressure product. Diastole is also a highly energetically demanding process,^41,42^ and more susceptible to ATP shortage than systole,^27^ hence, an improvement in diastolic function was seen with both –dp/dt and tau improving during Intralipid.

### Myocardial Oxygenation

When oxygen is limited, the disadvantage that FAO is inherently less oxygen-efficient becomes apparent (palmitate requires 23 O_2_ molecules to produce 105 ATP [P/O 2.3], whereas glucose requires only 6 O_2_ molecules to produce 31 ATP [P/O ≈2.5]).^43,44^ The lower P/O ratio of FAO accompanied by higher contractility (and therefore greater ATP demand) will increase myocardial oxygen use (MVo_2_), whereas cardiac efficiency (cardiac work per unit oxygen consumption) will be reduced. This can be seen in this study with higher MVo_2_ during Intralipid infusion, and during stress, and lower cardiac oxygen efficiency. However, as inotropes such as dobutamine are associated with increased death rate,^39,40^ it is important that the increased ATP demand during increased inotropy is met by oxidative metabolism. The phosphocreatine pool was not depleted, strongly implying that myocardial ATP demand did not exceed mitochondrial ATP supply.

Although we did not maximally exercise participants to the onset of ischemia, at 65% maximal heart rate, there was no evidence of insufficient oxygen supply to meet demand (no systolic dysfunction, PCr level not depleted, and overall cardiac lactate consumption). This would suggest that sufficient oxygen was available for normal mitochondrial oxidative phosphorylation, and that the mitochondrial oxygen supply was not exceeded during stress.

### The Effect of Additional Substrates

Recent work highlights 2 additional potential metabolic substrates: ketones and short-chain fatty acids. Despite low circulating levels (<2% of fasted substrate), ketones are readily taken up and metabolized,^45^ and have theoretical metabolic advantages: greater reduction of complex I of the electron transport chain and increased succinate,^46^ increasing the redox span between electron transport chain sites I and III, and the free energy for ATP synthesis.^47^ In this study, a trend to higher succinate production was seen during Intralipid infusion, during which ketones were taken up by the heart. Murine^6^ and human studies show increased β-hydroxybutyrate uptake in HF,^48^ which has been linked to increased stroke volume and MVo_2_ without change in oxygen efficiency when compared with an I+G infusion.^49^ However, despite an increase in β-hydroxybutyrate uptake during Intralipid infusion (0% to 6.7% of total substrate uptake; Figure 2B), as the molar yield of ATP of β-hydroxybutyrate is low (≈22.5/mole oxidized) when compared with FAO (≈120/mole oxidized), this would only be responsible for a very small proportion of ATP production. In addition, as the RQ when β-hydroxybutyrate is the sole substrate oxidized is ≈0.9,^50^ given the RQ recorded during intralipid infusion was 0.78, it is not likely that ketone was the primary substrate oxidized. Hence, although ketones undoubtedly have metabolic advantages, they are very unlikely to be responsible for the large increase in contractility seen during Intralipid infusion. Short-chain fatty acids (butyrate and isobutyrate) and branched-chain amino acids (valine, isoleucine, and leucine), although alternative energy substrates, did not show differences in uptake between infusions (data not shown), suggesting that these were not responsible for the contractile differences seen here.

### Toxic-Intermediary Metabolism

Although this study has shown that promoting FAO has advantages energetically and functionally in nonischemic HFrEF, this was achieved by means of increased LCFA delivery. Whereas this was necessary to overcome reduced fatty acid transport protein (CD36 [fatty acid transport protein]/FATP [fatty acid transport protein]) and CPT-1/2 transporter expression, the increasing intracellular pool of long-chain fatty acyl-CoA produces a larger pool of fatty acid substrate for nonoxidative processes, including the synthesis of triacylglycerol, diacylglycerol, and ceramides, which, in animal models, cause lipotoxicty.^51^ This study highlights that excessive delivery of fatty acids results in ceramide species being produced, and as a result, is unlikely to be a long-term solution to chronic HF. Likewise, increased sorbitol production during I+G infusion suggests polyol pathway activation. In general, balancing uptake and oxidation of any substrate to prevent spillover into toxic overflow pathways should be the goal of any therapy aimed at manipulating substrate supply.

### Limitations

This is a short-term study taking place over hours aimed to demonstrate the principle of substrate flexibility in the failing heart. These techniques to manipulate metabolic substrate supply may have limitations of lipotoxicity or insulin resistance. Other strategies are likely to be required for longer-term treatments to target fat metabolism directly.

Despite a history of HFrEF, elevated NT-proBNP (N-terminal pro–B-type natriuretic peptide), and echocardiographic evidence of LVEF <40% at screening, 2 participants had an LVEF >40% at baseline. This may be related to partial recovery of LVEF between screening and baseline. Despite this, the trend in response to metabolic intervention was similar in these 2 participants. Although it is a major cause of HF, ischemic cardiomyopathy was not included in this study, as the ^31^P spectroscopic techniques require homogenous myocardium.

To limit the time in which the participants underwent additional left coronary artery catheterization, flow wire, coronary sinus catheter, and LV catheter instrumentation during their cardiac resynchronization therapy implantation, and remove the potential for hypoglycemia, all participants underwent Intralipid infusion after the euglycemic clamp. Although this has the potential for systematic bias, we are confident that the primary objective of the infusion, to deliver excess substrate to the myocardium and delineate residual flexibility, was demonstrated.

Whereas cross-heart arteriovenous sampling allows uptake and efflux of metabolites, endogenous substrate flux is not measured directly.

### Conclusions

This study has shown that in compensated nonischemic cardiomyopathy with severe LV systolic dysfunction, the human heart retains substantial metabolic substrate flexibility if arterial supply or workload is changed. It also provides evidence that the heart is not oxygen-limited to a level that interferes with mitochondrial oxidative phosphorylation. In addition, it has been shown that increasing fatty acid uptake and oxidation, but not glucose, is associated with positive energetic and inotropic effects, with ATP production and consumption matched. There was no evidence of increased oxygen demand causing hypoxia. These findings challenge the existing hypotheses underlying metabolic modulator therapy in HF and suggest that increasing FAO is a promising therapeutic approach.

## Article Information

### Sources of Funding

This study was supported by British Heart Foundation clinical research training fellowships awarded to Drs Watson (FS/17/48/32907) and Green (FS/18/50/33807). Dr Rodgers is supported by the National Institute for Health and Care Research Cambridge Biomedical Research Centre (BRC-1215–20014). The views expressed are those of the authors and not necessarily those of the National Institute for Health and Care Research or the Department of Health and Social Care. Dr Valkovič is funded by a Sir Henry Dale fellowship awarded jointly by the Wellcome Trust and the Royal Society (221805/Z/20/Z) and supported by the Slovak Grant Agencies VEGA (2/0003/20) and APVV (19-0032). Drs Herring and Rider are supported by British Heart Foundation senior clinical research fellowships (FS/SCRF/20/32005 and FS/SCRF/22/32014).

### Disclosures

None.

### Supplemental Material

Expanded Methods

Figures S1–S4

Tables S1–S4

References 52–54

## Supplementary Material



## References

[1] Peterzan MA Lygate CA Neubauer S Rider OJ. Metabolic remodeling in hypertrophied and failing myocardium: a review. Am J Physiol Heart Circ Physiol. 2017;313:H597–H616. doi: 10.1152/ajpheart.00731.2016 28646030 10.1152/ajpheart.00731.2016

[2] Doenst T Nguyen TD Abel ED. Cardiac metabolism in heart failure: implications beyond ATP production. Circ Res. 2013;113:709–724. doi: 10.1161/CIRCRESAHA.113.300376 23989714 10.1161/CIRCRESAHA.113.300376 PMC3896379

[3] Heather LC Cole MA Lygate CA Evans RD Stuckey DJ Murray AJ Neubauer S Clarke K. Fatty acid transporter levels and palmitate oxidation rate correlate with ejection fraction in the infarcted rat heart. Cardiovasc Res. 2006;72:430–437. doi: 10.1016/j.cardiores.2006.08.020 17034771 10.1016/j.cardiores.2006.08.020

[4] Osorio JC Stanley WC Linke A Castellari M Diep QN Panchal AR Hintze TH Lopaschuk GD Recchia FA. Impaired myocardial fatty acid oxidation and reduced protein expression of retinoid X receptor-alpha in pacing-induced heart failure. Circulation. 2002;106:606–612. doi: 10.1161/01.cir.0000023531.22727.c1 12147544 10.1161/01.cir.0000023531.22727.c1

[5] Davila-Roman VG Vedala G Herrero P de las Fuentes L Rogers JG Kelly DP Gropler RJ. Altered myocardial fatty acid and glucose metabolism in idiopathic dilated cardiomyopathy. J Am Coll Cardiol. 2002;40:271–277. doi: 10.1016/s0735-1097(02)01967-8 12106931 10.1016/s0735-1097(02)01967-8

[6] Bedi KC Jr Snyder NW Brandimarto J Aziz M Mesaros C Worth AJ Wang LL Javaheri A Blair IA Margulies KB. Evidence for intramyocardial disruption of lipid metabolism and increased myocardial ketone utilization in advanced human heart failure. Circulation. 2016;133:706–716. doi: 10.1161/CIRCULATIONAHA.115.017545 26819374 10.1161/CIRCULATIONAHA.115.017545 PMC4779339

[7] Lahey R Wang X Carley AN Lewandowski ED. Dietary fat supply to failing hearts determines dynamic lipid signaling for nuclear receptor activation and oxidation of stored triglyceride. Circulation. 2014;130:1790–1799. doi: 10.1161/CIRCULATIONAHA.114.011687 25266948 10.1161/CIRCULATIONAHA.114.011687 PMC4229424

[8] Opie LH Knuuti J. The adrenergic-fatty acid load in heart failure. J Am Coll Cardiol. 2009;54:1637–1646. doi: 10.1016/j.jacc.2009.07.024 19850204 10.1016/j.jacc.2009.07.024

[9] Goldberg IJ Trent CM Schulze PC. Lipid metabolism and toxicity in the heart. Cell Metab. 2012;15:805–812. doi: 10.1016/j.cmet.2012.04.006 22682221 10.1016/j.cmet.2012.04.006 PMC3387529

[10] Flam E Jang C Murashige D Yang Y Morley MP Jung S Kantner DS Pepper H Bedi KC Jr Brandimarto J. Integrated landscape of cardiac metabolism in end-stage human nonischemic dilated cardiomyopathy. Nat Cardiovasc Res. 2022;1:817–829. doi: 10.1038/s44161-022-00117-6 36776621 10.1038/s44161-022-00117-6 PMC9910091

[11] Neglia D De Caterina A Marraccini P Natali A Ciardetti M Vecoli C Gastaldelli A Ciociaro D Pellegrini P Testa R. Impaired myocardial metabolic reserve and substrate selection flexibility during stress in patients with idiopathic dilated cardiomyopathy. Am J Physiol Heart Circ Physiol. 2007;293:H3270–H3278. doi: 10.1152/ajpheart.00887.2007 17921325 10.1152/ajpheart.00887.2007

[12] Brown DA Perry JB Allen ME Sabbah HN Stauffer BL Shaikh SR Cleland JG Colucci WS Butler J Voors AA. Expert consensus document: mitochondrial function as a therapeutic target in heart failure. Nat Rev Cardiol. 2017;14:238–250. doi: 10.1038/nrcardio.2016.203 28004807 10.1038/nrcardio.2016.203 PMC5350035

[13] Schroeder MA Lau AZ Chen AP Gu Y Nagendran J Barry J Hu X Dyck JR Tyler DJ Clarke K. Hyperpolarized (13)C magnetic resonance reveals early- and late-onset changes to in vivo pyruvate metabolism in the failing heart. Eur J Heart Fail. 2013;15:130–140. doi: 10.1093/eurjhf/hfs192 23258802 10.1093/eurjhf/hfs192 PMC3547367

[14] Zhang L Jaswal JS Ussher JR Sankaralingam S Wagg C Zaugg M Lopaschuk GD. Cardiac insulin-resistance and decreased mitochondrial energy production precede the development of systolic heart failure after pressure-overload hypertrophy. Circ Heart Fail. 2013;6:1039–1048. doi: 10.1161/CIRCHEARTFAILURE.112.000228 23861485 10.1161/CIRCHEARTFAILURE.112.000228

[15] Shibayama J Yuzyuk TN Cox J Makaju A Miller M Lichter J Li H Leavy JD Franklin S Zaitsev AV. Metabolic remodeling in moderate synchronous versus dyssynchronous pacing-induced heart failure: integrated metabolomics and proteomics study. PLoS One. 2015;10:e0118974. doi: 10.1371/journal.pone.0118974 25790351 10.1371/journal.pone.0118974 PMC4366225

[16] Ritterhoff J Young S Villet O Shao D Neto FC Bettcher LF Hsu Y-WA Kolwicz SC Jr Raftery D Tian R. Metabolic remodeling promotes cardiac hypertrophy by directing glucose to aspartate biosynthesis. Circ Res. 2020;126:182–196. doi: 10.1161/circresaha.119.315483 31709908 10.1161/CIRCRESAHA.119.315483 PMC8448129

[17] Lopaschuk GD. Metabolic modulators in heart disease: past, present, and future. Can J Cardiol. 2017;33:838–849. doi: 10.1016/j.cjca.2016.12.013 28279520 10.1016/j.cjca.2016.12.013

[18] Lopaschuk GD Rebeyka IM Allard MF. Metabolic modulation: a means to mend a broken heart. Circulation. 2002;105:140–142. 11790689

[19] Sankaralingam S Lopaschuk GD. Cardiac energy metabolic alterations in pressure overload-induced left and right heart failure (2013 Grover Conference Series). Pulm Circ. 2015;5:15–28. doi: 10.1086/679608 25992268 10.1086/679608 PMC4405723

[20] Zolk O Solbach TF Eschenhagen T Weidemann A Fromm MF. Activation of negative regulators of the hypoxia-inducible factor (HIF) pathway in human end-stage heart failure. Biochem Biophys Res Commun. 2008;376:315–320. doi: 10.1016/j.bbrc.2008.08.152 18782560 10.1016/j.bbrc.2008.08.152

[21] Bell SP Adkisson DW Ooi H Sawyer DB Lawson MA Kronenberg MW. Impairment of subendocardial perfusion reserve and oxidative metabolism in nonischemic dilated cardiomyopathy. J Card Fail. 2013;19:802–810. doi: 10.1016/j.cardfail.2013.10.010 24331202 10.1016/j.cardfail.2013.10.010 PMC3945036

[22] Liao R Jain M Cui L D’Agostino J Aiello F Luptak I Ngoy S Mortensen RM Tian R. Cardiac-specific overexpression of GLUT1 prevents the development of heart failure attributable to pressure overload in mice. Circulation. 2002;106:2125–2131. doi: 10.1161/01.cir.0000034049.61181.f3 12379584 10.1161/01.cir.0000034049.61181.f3

[23] Kantor PF Lucien A Kozak R Lopaschuk GD. The antianginal drug trimetazidine shifts cardiac energy metabolism from fatty acid oxidation to glucose oxidation by inhibiting mitochondrial long-chain 3-ketoacyl coenzyme A thiolase. Circ Res. 2000;86:580–588. doi: 10.1161/01.res.86.5.580 10720420 10.1161/01.res.86.5.580

[24] Sabbah HN Chandler MP Mishima T Suzuki G Chaudhry P Nass O Biesiadecki BJ Blackburn B Wolff A Stanley WC. Ranolazine, a partial fatty acid oxidation (pFOX) inhibitor, improves left ventricular function in dogs with chronic heart failure. J Card Fail. 2002;8:416–422. doi: 10.1054/jcaf.2002.129232 12528095 10.1054/jcaf.2002.129232

[25] Lee L Campbell R Scheuermann-Freestone M Taylor R Gunaruwan P Williams L Ashrafian H Horowitz J Fraser AG Clarke K. Metabolic modulation with perhexiline in chronic heart failure: a randomized, controlled trial of short-term use of a novel treatment. Circulation. 2005;112:3280–3288. doi: 10.1161/CIRCULATIONAHA.105.551457 16301359 10.1161/CIRCULATIONAHA.105.551457

[26] Schmidt-Schweda S Holubarsch C. First clinical trial with etomoxir in patients with chronic congestive heart failure. Clin Sci (Lond). 2000;99:27–35. 10887055

[27] Jatain S Kapoor A Sinha A Khanna R Kumar S Garg N Tewari S Goel P. Metabolic manipulation in dilated cardiomyopathy: assessing the role of trimetazidine. Indian Heart J. 2016;68:803–808. doi: 10.1016/j.ihj.2016.04.023 27931551 10.1016/j.ihj.2016.04.023 PMC5143816

[28] Fragasso G Perseghin G De Cobelli F Esposito A Palloshi A Lattuada G Scifo P Calori G Del Maschio A Margonato A. Effects of metabolic modulation by trimetazidine on left ventricular function and phosphocreatine/adenosine triphosphate ratio in patients with heart failure. Eur Heart J. 2006;27:942–948. doi: 10.1093/eurheartj/ehi816 16510466 10.1093/eurheartj/ehi816

[29] Taylor M Wallhaus TR Degrado TR Russell DC Stanko P Nickles RJ Stone CK. An evaluation of myocardial fatty acid and glucose uptake using PET with [18F]fluoro-6-thia-heptadecanoic acid and [18F]FDG in patients with congestive heart failure. J Nucl Med. 2001;42:55–62. 11197981

[30] Swan JW Anker SD Walton C Godsland IF Clark AL Leyva F Stevenson JC Coats AJ. Insulin resistance in chronic heart failure: relation to severity and etiology of heart failure. J Am Coll Cardiol. 1997;30:527–532. doi: 10.1016/s0735-1097(97)00185-x 9247528 10.1016/s0735-1097(97)00185-x

[31] Ponikowski P Voors AA Anker SD Bueno H Cleland JGF Coats AJS Falk V Gonzalez-Juanatey JR Harjola VP Jankowska EA; ESC Scientific Document Group. 2016 ESC Guidelines for the diagnosis and treatment of acute and chronic heart failure. Eur Heart J. 2016;37:2129–2200. doi: 10.1093/eurheartj/ehw12827206819

[32] Rider OJ Francis JM Ali MK Byrne J Clarke K Neubauer S Petersen SE. Determinants of left ventricular mass in obesity; a cardiovascular magnetic resonance study. J Cardiovasc Magn Reson. 2009;11:9. doi: 10.1186/1532-429X-11-9 19393079 10.1186/1532-429X-11-9 PMC2680851

[33] Watson WD Timm KN Lewis AJ Miller JJJ Emmanuel Y Clarke K Neubauer S Tyler DJ Rider OJ. Nicotinic acid receptor agonists impair myocardial contractility by energy starvation. FASEB J. 2020;34:14878–14891. doi: 10.1096/fj.202000084rr 32954525 10.1096/fj.202000084RR

[34] Purvis LAB Clarke WT Biasiolli L Valkovic L Robson MD Rodgers CT. OXSA: An open-source magnetic resonance spectroscopy analysis toolbox in MATLAB. PLoS One. 2017;12:e0185356. doi: 10.1371/journal.pone.0185356 28938003 10.1371/journal.pone.0185356 PMC5609763

[35] Clarke WT Peterzan MA Rayner JJ Sayeed RA Petrou M Krasopoulos G Lake HA Raman B Watson WD Cox P. Localized rest and stress human cardiac creatine kinase reaction kinetics at 3 T. NMR Biomed. 2019;e4085. doi: 10.1002/nbm.4085 30920054 10.1002/nbm.4085 PMC6542687

[36] Weiss RG Gerstenblith G Bottomley PA. ATP flux through creatine kinase in the normal, stressed, and failing human heart. Proc Natl Acad Sci USA. 2005;102:808–813. doi: 10.1073/pnas.0408962102 15647364 10.1073/pnas.0408962102 PMC545546

[37] Mookerjee SA Gerencser AA Nicholls DG Brand MD. Quantifying intracellular rates of glycolytic and oxidative ATP production and consumption using extracellular flux measurements. J Biol Chem. 2017;292:7189–7207. doi: 10.1074/jbc.M116.774471 28270511 10.1074/jbc.M116.774471 PMC5409486

[38] Karwi QG Uddin GM Ho KL Lopaschuk GD. Loss of metabolic flexibility in the failing heart. Front Cardiovasc Med. 2018;5:68. doi: 10.3389/fcvm.2018.00068 29928647 10.3389/fcvm.2018.00068 PMC5997788

[39] Tarvasmäki T Lassus J Varpula M Sionis A Sund R Køber L Spinar J Parissis J Banaszewski M Silva Cardoso J. Current real-life use of vasopressors and inotropes in cardiogenic shock-adrenaline use is associated with excess organ injury and mortality. Crit Care. 2016;20:1–11. doi: 10.1186/s13054-016-1387-1 27374027 10.1186/s13054-016-1387-1 PMC4931696

[40] Maack C Eschenhagen T Hamdani N Heinzel FR Lyon AR Manstein DJ Metzger J Papp Z Tocchetti CG Yilmaz MB. Treatments targeting inotropy. Eur Heart J. 2019;40:3626–3644. doi: 10.1093/eurheartj/ehy600 30295807 10.1093/eurheartj/ehy600 PMC7963133

[41] Peterson LR Waggoner AD Schechtman KB Meyer T Gropler RJ Barzilai B Davila-Roman VG. Alterations in left ventricular structure and function in young healthy obese women: assessment by echocardiography and tissue Doppler imaging. J Am Coll Cardiol. 2004;43:1399–1404. doi: 10.1016/j.jacc.2003.10.062 15093874 10.1016/j.jacc.2003.10.062

[42] Otto ME Belohlavek M Khandheria B Gilman G Svatikova A Somers V. Comparison of right and left ventricular function in obese and nonobese men. Am J Cardiol. 2004;93:1569–1572. doi: 10.1016/j.amjcard.2004.02.073 15194042 10.1016/j.amjcard.2004.02.073

[43] How O-J Aasum E Kunnathu S Severson DL Myhre ESP Larsen TS. Influence of substrate supply on cardiac efficiency, as measured by pressure-volume analysis in ex vivo mouse hearts. Am J Physiol Heart Circ Physiol. 2005;288:H2979–H2985. doi: 10.1152/ajpheart.00084.2005 15764683 10.1152/ajpheart.00084.2005

[44] Burkhoff D Weiss RG Schulman SP Kalil-Filho R Wannenburg T Gerstenblith G. Influence of metabolic substrate on rat heart function and metabolism at different coronary flows. Am J Physiol. 1991;261:H741–H750. doi: 10.1152/ajpheart.1991.261.3.H741 1887921 10.1152/ajpheart.1991.261.3.H741

[45] Cotter DG Schugar RC Crawford PA. Ketone body metabolism and cardiovascular disease. Am J Physiol Heart Circ Physiol. 2013;304:H1060–H1076. doi: 10.1152/ajpheart.00646.2012 23396451 10.1152/ajpheart.00646.2012 PMC3625904

[46] Sato K Kashiwaya Y Keon CA Tsuchiya N King MT Radda GK Chance B Clarke K Veech RL. Insulin, ketone bodies, and mitochondrial energy transduction. FASEB J. 1995;9:651–658. doi: 10.1096/fasebj.9.8.7768357 7768357 10.1096/fasebj.9.8.7768357

[47] Veech RL. The therapeutic implications of ketone bodies: the effects of ketone bodies in pathological conditions: ketosis, ketogenic diet, redox states, insulin resistance, and mitochondrial metabolism. Prostaglandins Leukot Essent Fatty Acids. 2004;70:309–319. doi: 10.1016/j.plefa.2003.09.007 14769489 10.1016/j.plefa.2003.09.007

[48] Aubert G Martin OJ Horton JL Lai L Vega RB Leone TC Koves T Gardell SJ Kruger M Hoppel CL. The failing heart relies on ketone bodies as a fuel. Circulation. 2016;133:698–705. doi: 10.1161/CIRCULATIONAHA.115.017355 26819376 10.1161/CIRCULATIONAHA.115.017355 PMC4766035

[49] Nielsen R Moller N Gormsen LC Tolbod LP Hansson NH Sorensen J Harms HJ Frokiaer J Eiskjaer H Jespersen NR. Cardiovascular effects of treatment with the ketone body 3-hydroxybutyrate in chronic heart failure patients. Circulation. 2019;139:2129–2141. doi: 10.1161/CIRCULATIONAHA.118.036459 30884964 10.1161/CIRCULATIONAHA.118.036459 PMC6493702

[50] Frayn KN. Calculation of substrate oxidation rates in vivo from gaseous exchange. J Appl Physiol Respir Environ Exerc Physiol. 1983;55:628–634. doi: 10.1152/jappl.1983.55.2.628 6618956 10.1152/jappl.1983.55.2.628

[51] Chiu HC Kovacs A Ford DA Hsu FF Garcia R Herrero P Saffitz JE Schaffer JE. A novel mouse model of lipotoxic cardiomyopathy. J Clin Invest. 2001;107:813–822. doi: 10.1172/JCI10947 11285300 10.1172/JCI10947 PMC199569

[52] Seemann F Arvidsson P Nordlund D Kopic S Carlsson M Arheden H Heiberg E. Noninvasive quantification of pressure-volume loops from brachial pressure and cardiovascular magnetic resonance. Circ Cardiovasc Imaging. 2019;12:e008493. doi: 10.1161/CIRCIMAGING.118.008493 30630347 10.1161/CIRCIMAGING.118.008493

[53] Senzaki H Chen CH Kass DA. Single-beat estimation of end-systolic pressure-volume relation in humans: a new method with the potential for noninvasive application. Circulation. 1996;94:2497–2506. doi: 10.1161/01.cir.94.10.2497 8921794 10.1161/01.cir.94.10.2497

[54] Sjoberg P Seemann F Arheden H Heiberg E. Non-invasive quantification of pressure-volume loops from cardiovascular magnetic resonance at rest and during dobutamine stress. Clin Physiol Funct Imaging. 2021;41:467–470. doi: 10.1111/cpf.12718 34121316 10.1111/cpf.12718

