# Supplementary material for: Retained Metabolic Flexibility of the Failing Human Heart
Source: Circulation. 2023 May 18;148(2):109–23. doi: 10.1161/CIRCULATIONAHA.122.062166 (PMC10417210; doi:10.1161/CIRCULATIONAHA.122.062166)
Supplement: Supplementary file 1 [file cir-148-109-s001.pdf]

SUPPLEMENTAL MATERIAL

Expanded Methods

Validation of CMR Derived Pressure Volume Loop from CMR and Blood pressure

Recent developments have enabled non-invasively computed PV loop measurements based on CMR volumetry and a brachial blood pressure measurement[52]. Using an algorithm trained on invasive pressure data from a porcine model, this approach combines LV volumes with a time-varying elastance function to compute time-resolved LV pressures[53]. While this algorithm has been validated using invasive data from a porcine model and sensitivity tested using a dobutamine infusion in healthy humans[54], we here validated its use for quantification of stroke work and end-systolic pressure-volume relationship in patients using invasive left ventricular pressure recordings.

To validate the performance of non-invasively computed PV loops against invasive measures in human patients, four heart failure patients underwent two subsequent sessions of CMR cine imaging and simultaneous brachial blood pressure measurement, with intravenous administration of two different metabolic substrate infusions (Intralipid and insulin+glucose), producing two different hemodynamic states for each patient. LV catheterization was then conducted with sequential administration of the same metabolic substrates. Pressure-volume loops were computed from CMR volumes combined with 1) a time-varying elastance function scaled to brachial blood pressure and temporally stretched to match volume data, and 2) invasive pressures

averaged from multiple sampled beats. Method comparison was conducted using linear regression and Bland-Altman analysis.

The Figure below shows non-invasively derived PV loop parameters compared to invasive data. The non-invasive method demonstrated strong correlations and low bias for stroke work (R^2=0.97, bias 4.6%, p<0.0001), and end-systolic pressure-volume relationship (R^2=0.90, bias 5.4%, p=0.0003).

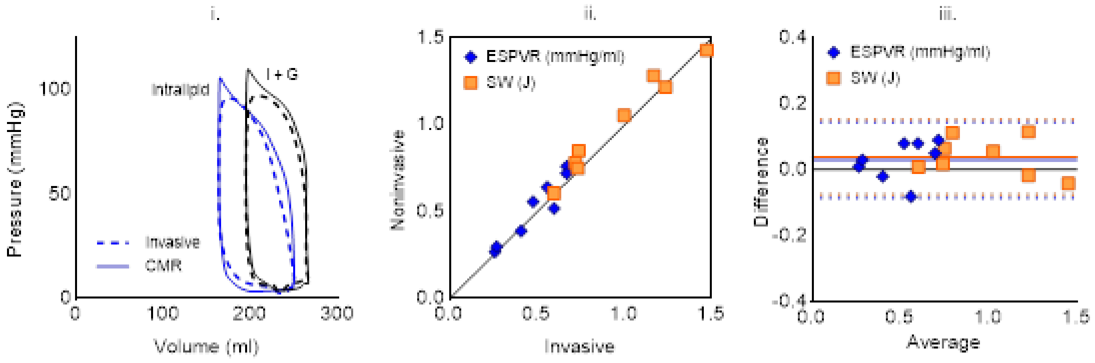

<u>Figure S1</u>. Left: Pressure-volume loops in one patient at two different hemodynamic states (blue during intralipid and black during glucose + insulin infusion). Solid line: data computed using non-invasive method, broken line: invasive method. ii) Scatter plot for the studied pressure-volume loop parameters. Solid lines indicate the lines of identity. ESPVR, end-systolic pressure-volume relationship, iii) Bland-Altman method comparison showing bias and limits of agreement for ESPVR and SW.

	R^2	Bias	LoA (%)
Stroke work	0.97	4.6	11.5
ESPVR	0.90	5.4	20.6

Table S1. Coefficients of determination, bias and limits of agreement (LoA) for the different parameters.

Following validation, this was applied to the non-invasive data to determine LV stroke work and ESPVR during substrate manipulation.

Metabolomics

All metabolomics were run by a commercial provider (Metabolon, Durham NC), USA and analyzed as follows. Following receipt, samples were stored at -80°C. The samples were prepared with an automated system (MicroLab STAR, Hamilton Company, Reno, NV). Several recovery standards were added prior to the first step in the extraction process for QC purposes. To remove protein, dissociate small molecules bound to protein or trapped in the precipitated protein matrix, and to recover chemically diverse metabolites, proteins were precipitated with methanol under vigorous shaking for 2 min (Glen Mills GenoGrinder 2000) followed by centrifugation. The resulting extract was divided into five fractions: two for analysis by two separate reverse phases (RP)/UPLC-MS/MS methods with positive ion mode electrospray

ionization (ESI), one for analysis by RP/UPLC-MS/MS with negative ion mode ESI, one for analysis by HILIC/UPLC-MS/MS with negative ion mode ESI, and one sample was reserved for backup. Samples were placed briefly on a TurboVap® (Zymark) to remove the organic solvent. The sample extracts were stored overnight under nitrogen before preparation for analysis. QA/QC: Several types of controls were analyzed in concert with the experimental samples: a pooled matrix sample generated by taking a small volume of each experimental sample (or alternatively, use of a pool of well-characterized human plasma) served as a technical replicate throughout the data set; extracted water samples served as process blanks; and a cocktail of QC standards that were carefully chosen not to interfere with the measurement of endogenous compounds were spiked into every analyzed sample, allowed instrument performance monitoring and aided chromatographic alignment.

Ultrahigh Performance Liquid Chromatography-Tandem Mass Spectroscopy (UPLC-MS/MS): All methods utilized a Waters ACQUITY ultra-performance liquid chromatography (UPLC) and a Thermo Scientific Q-Exactive high resolution/accurate mass spectrometer interfaced with a heated electrospray ionization (HESI-II) source and Orbitrap mass analyzer operated at 35,000 mass resolution. The sample extract was dried then reconstituted in solvents compatible to each of the four methods. Each reconstitution solvent contained a series of standards at fixed concentrations to ensure injection and chromatographic consistency. One aliquot was analyzed using acidic positive ion conditions, chromatographically optimized for more hydrophilic compounds. In this method, the extract was gradient eluted from a C18 column (Waters UPLC BEH C18-2.1x100 mm, 1.7 µm) using water and methanol, containing

0.05% perfluoropentanoic acid (PFPA) and 0.1% formic acid (FA). Another aliquot was also analyzed using acidic positive ion conditions, however it was chromatographically optimized for more hydrophobic compounds. In this method, the extract was gradient eluted from the same afore mentioned C18 column using methanol, acetonitrile, water, 0.05% PFPA and 0.01% FA and was operated at an overall higher organic content. Another aliquot was analyzed using basic negative ion optimized conditions using a separate dedicated C18 column. The basic extracts were gradient eluted from the column using methanol and water, however with 6.5mM Ammonium Bicarbonate at pH 8. The fourth aliquot was analyzed via negative ionization following elution from a HILIC column (Waters UPLC BEH Amide 2.1x150 mm, 1.7 µm) using a gradient consisting of water and acetonitrile with 10mM Ammonium Formate, pH 10.8. The MS analysis alternated between MS and data-dependent MS^n scans using dynamic exclusion. The scan range varied slighted between methods but covered 70-1000 m/z. Raw data files are archived and extracted as described below. Data Extraction and Compound Identification and Quantification Raw data was extracted, peak-identified and QC processed using Metabolon's hardware and software. Compounds were identified by comparison to library entries of purified standards or recurrent unknown entities.

Metabolite Quantification and Data Normalization: Peaks were quantified using area-under-the-curve. A data normalization step was performed to correct variation resulting from instrument inter-day tuning differences. Each compound was corrected in run-day blocks by registering the medians to equal one (1.00) and normalizing each data point proportionately.

Lipidomics

All Lipidomics analysis were run by a commercial provider (Metabolon, Durham NC), USA and as follows. Following receipt, samples were stored at -80oC. Lipids were extracted from the biofluid in the presence of deuterated internal standards using an automated BUME extraction according to the method of Lofgren et al. (J Lipid Res 2012:53(8):1690-700). The extracts were concentrated under nitrogen and reconstituted in 0.25mL of 10mM ammonium acetate dichloromethane:methanol (50:50). The extracts were transferred to inserts and placed in vials for infusion-MS analysis, performed on a Shimazdu LC with nano PEEK tubing and the Sciex SelexIon-5500 QTRAP. The samples were analyzed via both positive and negative mode electrospray. The 5500 QTRAP scan was performed in MRM mode with the total of more than 1,100 MRMs. Individual lipid species were quantified by taking the peak area ratios of target compounds and their assigned internal standards, then multiplying by the concentration of internal standard added to the sample. Lipid species concentrations were background-subtracted using the concentrations detected in process blanks (water extracts) and run day normalized (when applicable). The resulting background-subtracted, run-day normalized lipid species concentrations were then used to calculate the lipid class and fatty acid total concentrations, as well as the mol% composition values for lipid species, lipid classes, and fatty acids.

Supplemental Data

Table S2: Demographic Data for Healthy Volunteer Population

	Mean	Range
Sex Mix	3M:7F	
Age	41	24-79
Height (m)	1.69	1.57-1.92
Weight (kg)	67	53-85
BMI (kg/m^2)	23.4	20.6-27.1
Fasting venous blood glucose (mmol/L)	5.2	4.3 – 6.3

Table S3: Ceramide species breakdown during glucose-insulin infusion

	Glucose + Insulin				
	Left Mainstem		Coronary Sinus		CS/A
uM	Mean	SD	Mean	SD	Mean
CER[FA18:1]	0.0061	0.0021	0.0066	0.0019	1.0890
CER[FA22:0]	0.6583	0.2617	0.6329	0.2030	0.9614
CER[FA24:1]	0.9931	0.3046	0.9810	0.2437	0.9879
CER[FA26:1]	0.0192	0.0047	0.0190	0.0035	0.9898
CER[FA16:0]	0.2757	0.0838	0.2800	0.0779	1.0159
CER[FA20:1]	0.0049	0.0010	0.0054	0.0010	1.1002
CER[FA22:1]	0.0286	0.0088	0.0268	0.0062	0.9360
CER[FA18:0]	0.0998	0.0347	0.0984	0.0316	0.9858
CER[FA20:0]	0.0900	0.0275	0.0856	0.0224	0.9505
CER[FA24:0]	2.3778	1.4230	2.2259	1.0731	0.9361
CER[FA14:0]	0.0108	0.0046	0.0115	0.0036	1.0647
CER[FA26:0]	0.0291	0.0094	0.0284	0.0069	0.9776

Table S4: Ceramide species breakdown Intralipid Infusion

	Intralipid				
	Left Mainstem		Coronary Sinus		CS/A
uM	Mean	SD	Mean	SD	Mean
CER[FA18:1]	0.0075	0.0020	0.0071	0.0015	0.9395
CER[FA22:0]	0.7189	0.2077	0.7657	0.2382	1.0652
CER[FA24:1]	1.0647	0.2723	1.1074	0.2884	1.0401
CER[FA26:1]	0.0196	0.0048	0.0201	0.0046	1.0239
CER[FA16:0]	0.3164	0.0802	0.3435	0.0960	1.0858
CER[FA20:1]	0.0050	0.0013	0.0052	0.0012	1.0299
CER[FA22:1]	0.0353	0.0079	0.0360	0.0091	1.0191
CER[FA18:0]	0.1155	0.0351	0.1183	0.0334	1.0242
CER[FA20:0]	0.0964	0.0257	0.0994	0.0271	1.0306
CER[FA24:0]	2.2733	1.0032	2.4141	1.2314	1.0619
CER[FA14:0]	0.0104	0.0038	0.0112	0.0027	1.0841
CER[FA26:0]	0.0287	0.0070	0.0304	0.0091	1.0605

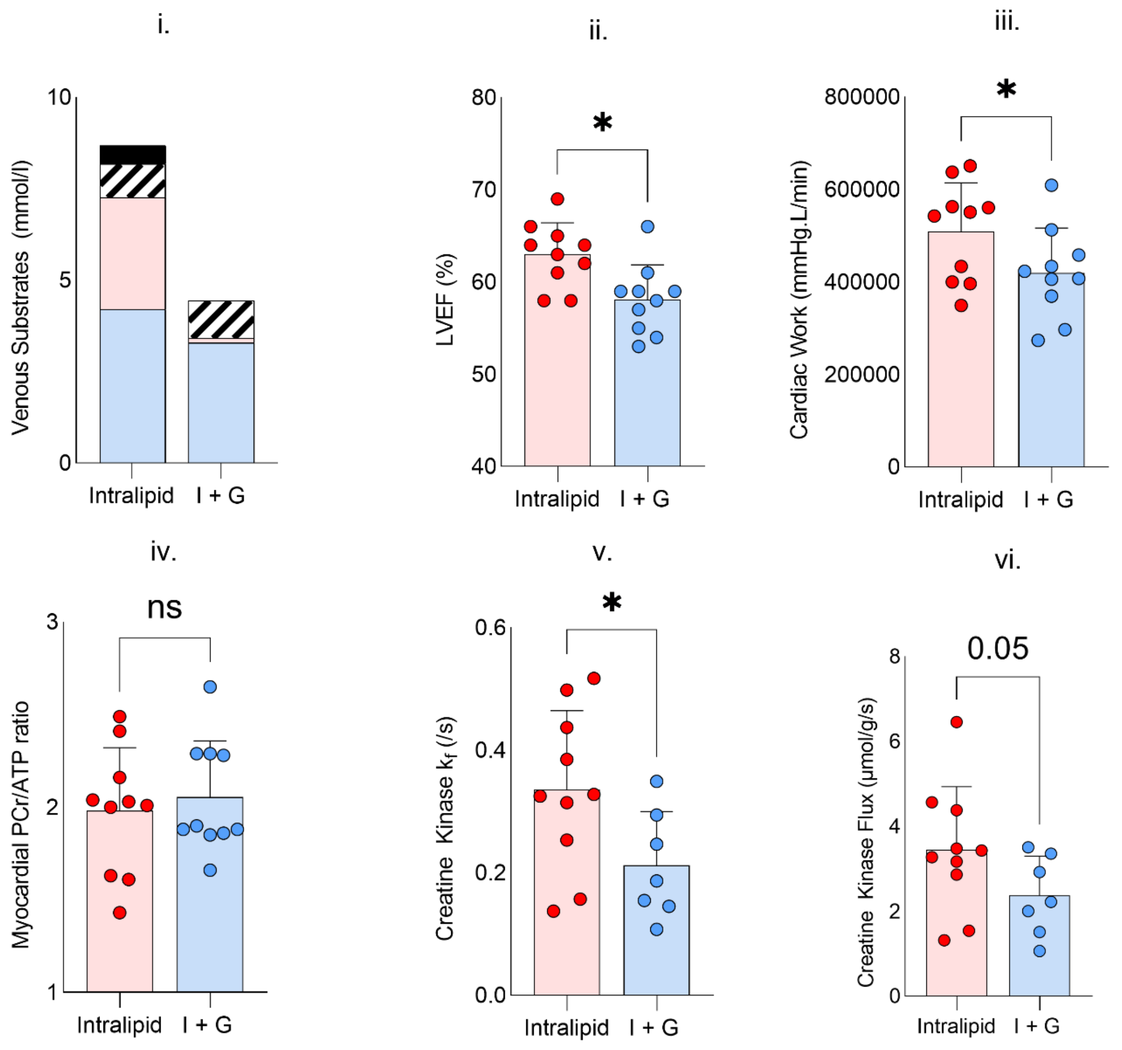

Figure S2. Data from healthy volunteers. i. Relative proportions of energetic substrates in peripheral venous blood, blue = glucose, red = free fatty acids, hatched = lactate, black = beta-hydroxybutyrate. ii. Left ventricular ejection fraction from magnetic resonance cine imaging. iii. Cardiac work calculated from magnetic resonance imaging and non-invasive blood pressure. iv. Myocardial phosphocreatine/ATP ratio. v. Creatine kinase pseudo first order rate constant. vi. Creatine Kinase Flux.

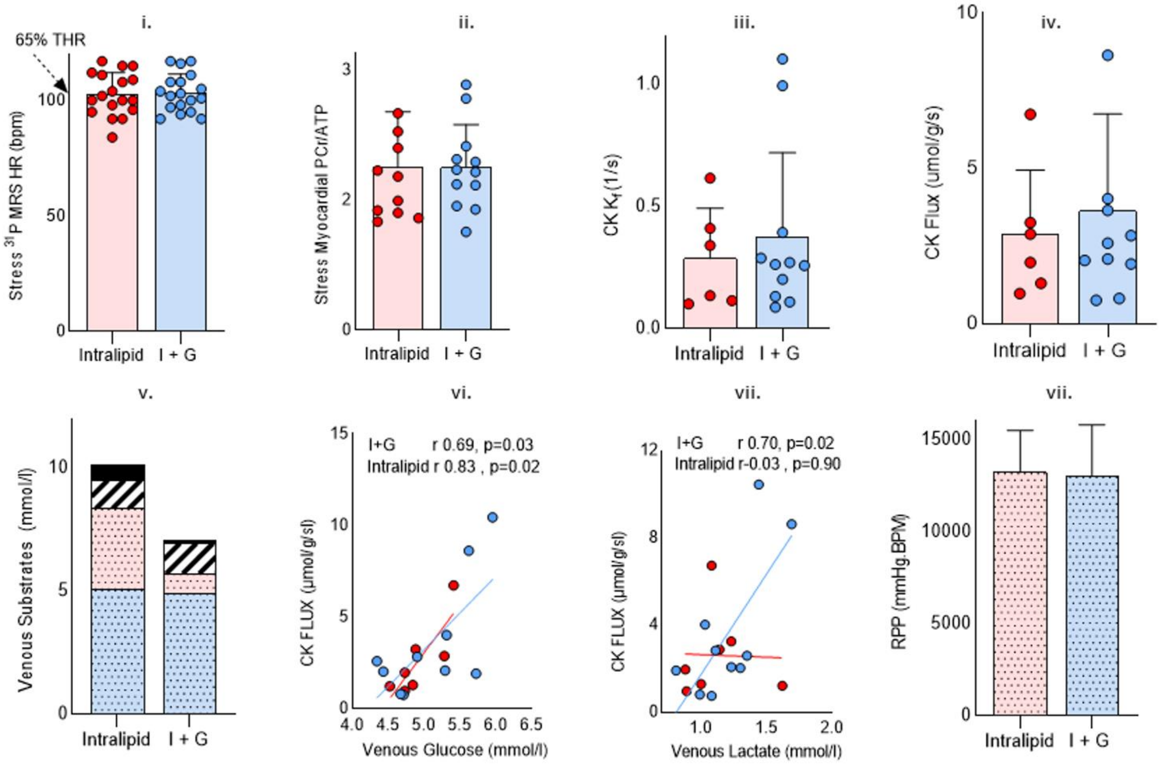

Figure S3. Assessment of myocardial energetics recorded during dobutamine infusion i) achieved heart rate, 65% target heart rate (THR) is highlighted. ii-iv) shows PCr/ATP, CK k_f, and CK flux, v) infusions specific venous circulating substrates during stress. The relationships between CK flux and venous glucose and lactate concentration are shown in panels vi) and vii). viii). RPP during stress. Spotted indicates stress measurement, * denotes $p<0.05$, Data is presented a mean with SD error bars.

Left Mainstem Arterial Sampling Data

A i ii iii iv

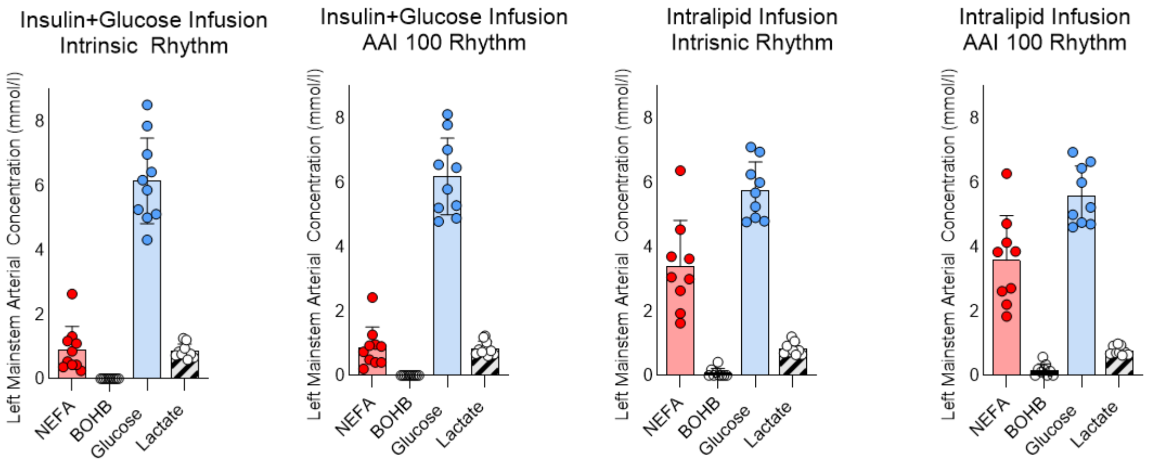

Coronary Sinus Vein Sampling Data

B i ii iii iv

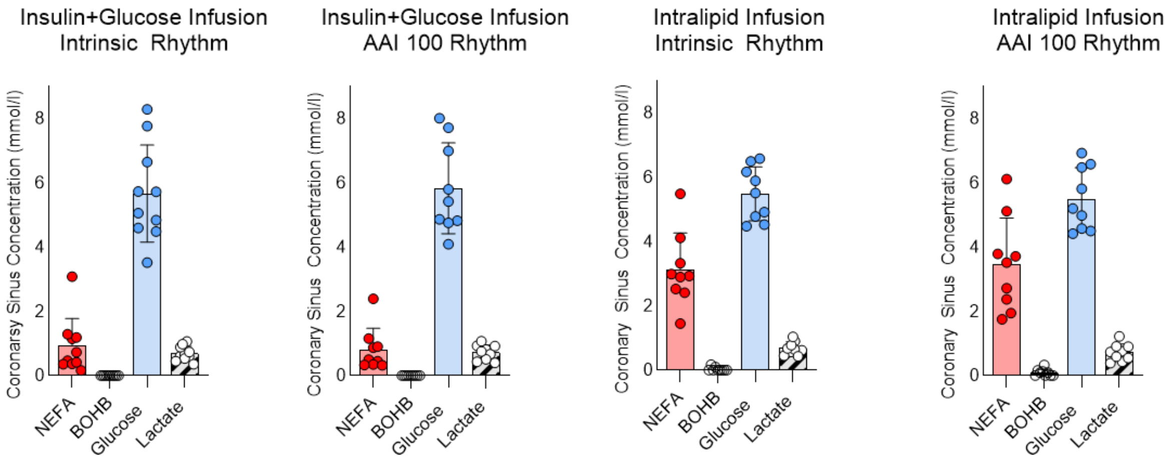

Calculated Left Mainstem - Coronary Sinus Differences

C i ii iii iv

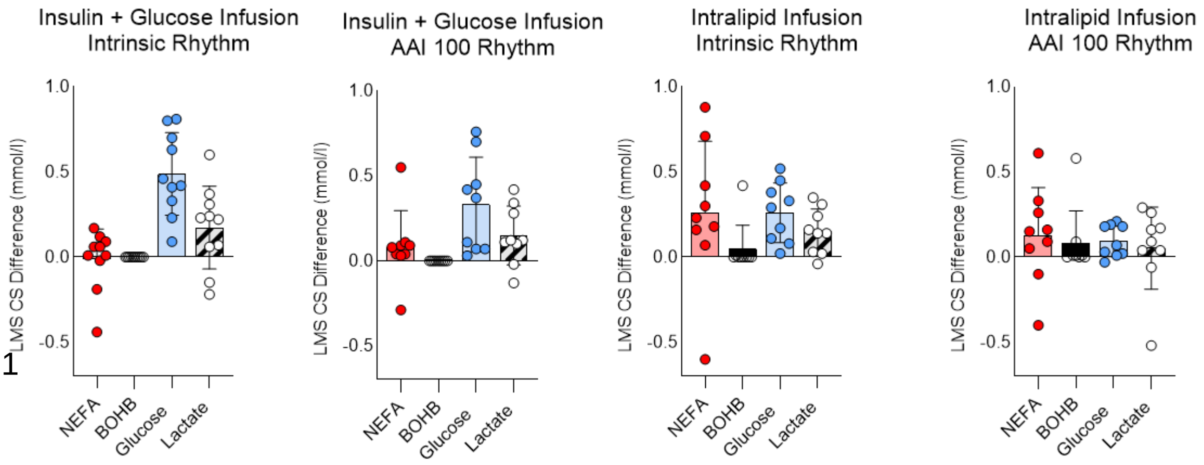

Figure S4: Break down of individual sampling datapoints to expand stacked bar charts from figures 1,2 and 3. Row A: substrate concentrations in mmol/L for non-esterified fatty acids (NEFA), beta-hydroxybutyrate (BOHB), glucose and lactate taken from left main coronary artery sampling. Row B: substrate concentrations taken from coronary sinus blood. Row C: arteriovenous concentration difference. In each row, (i) shows samples taken during intrinsic rhythm during euglycemic clamp, (ii) shows samples taken during rapid atrial pacing during euglycemic clamp, (iii) shows samples taken during intrinsic rhythm during intralipid infusion and (iv) shows samples taken during rapid atrial pacing during intralipid infusion.
